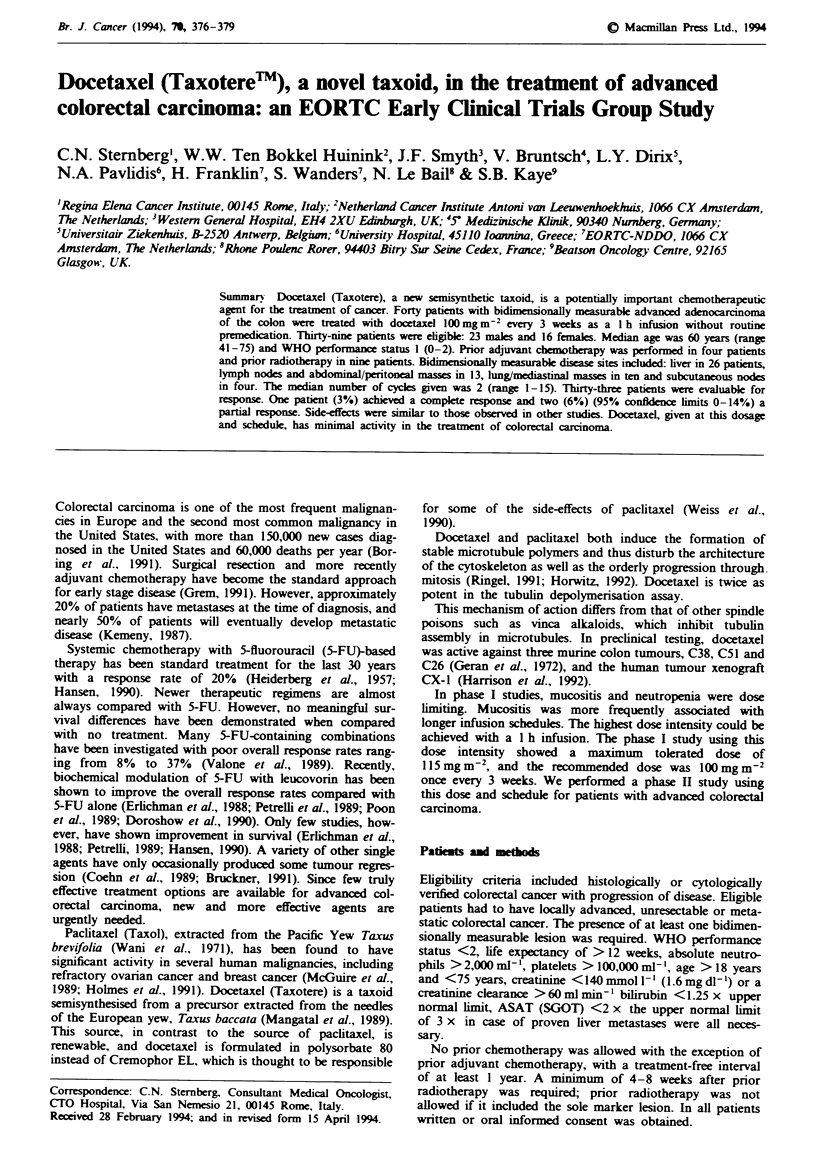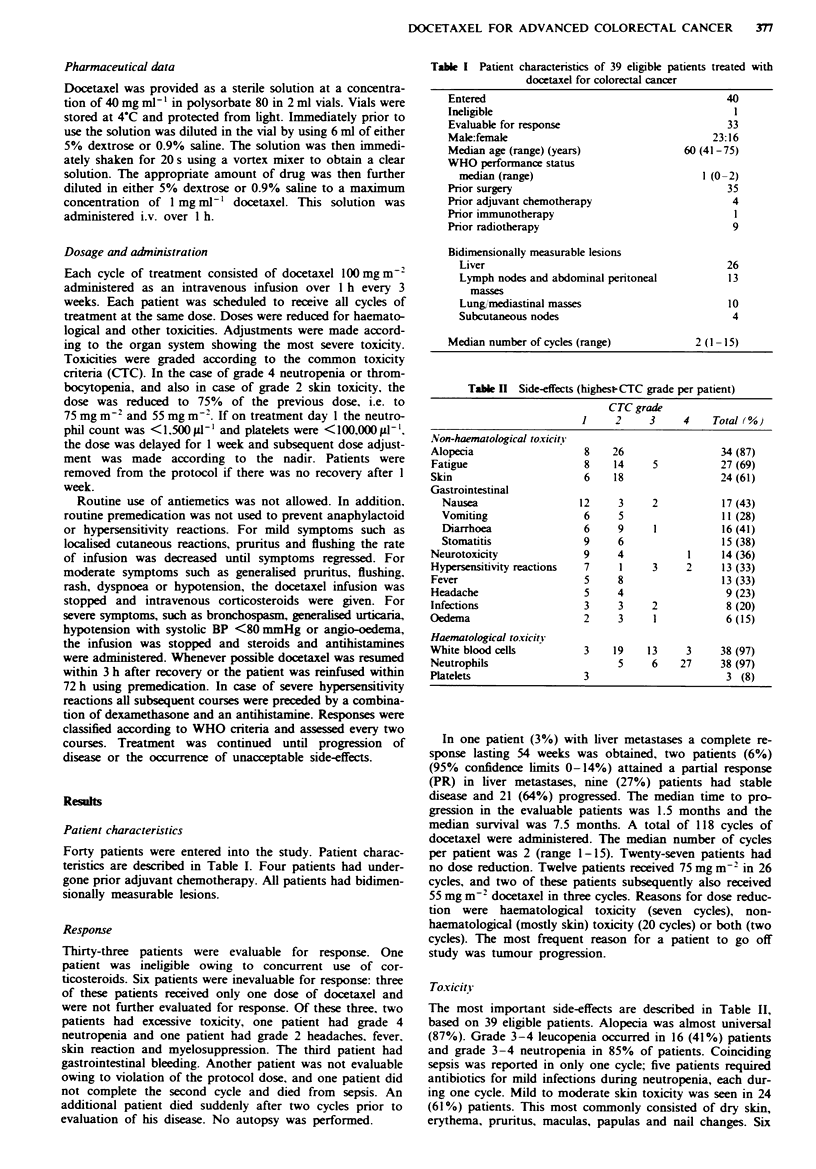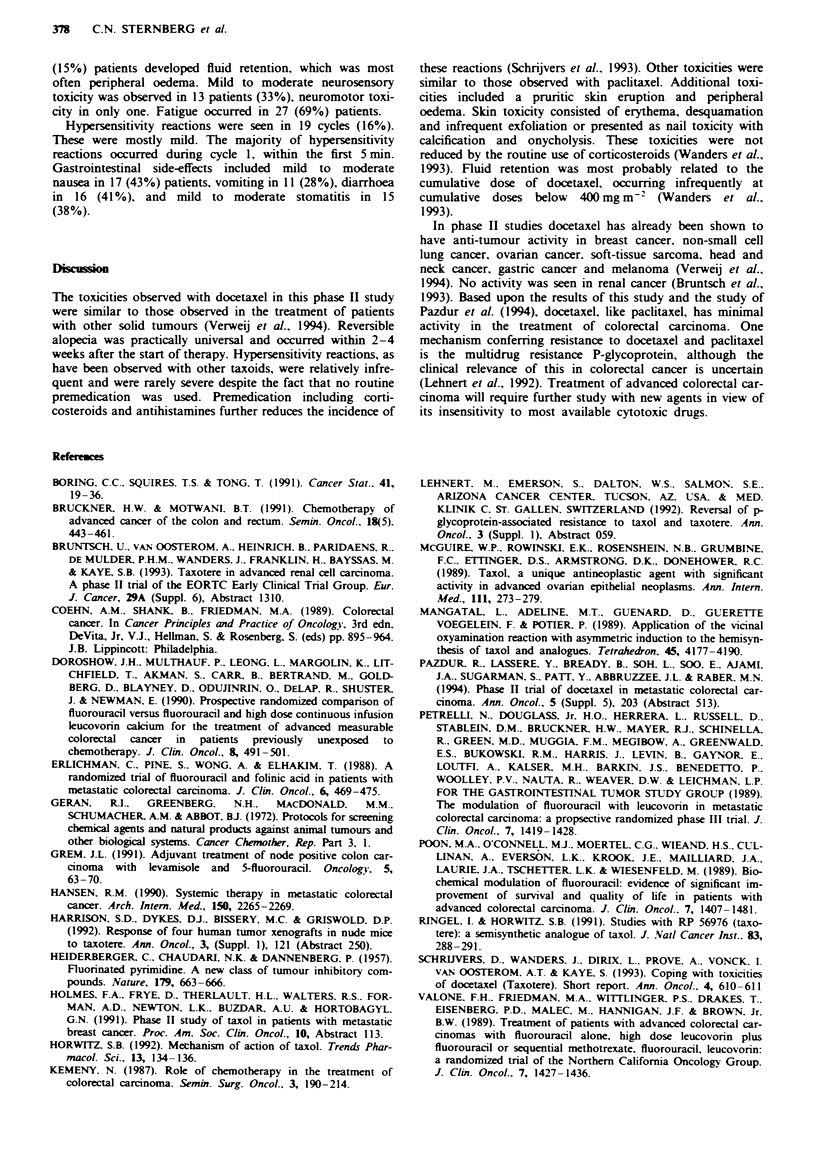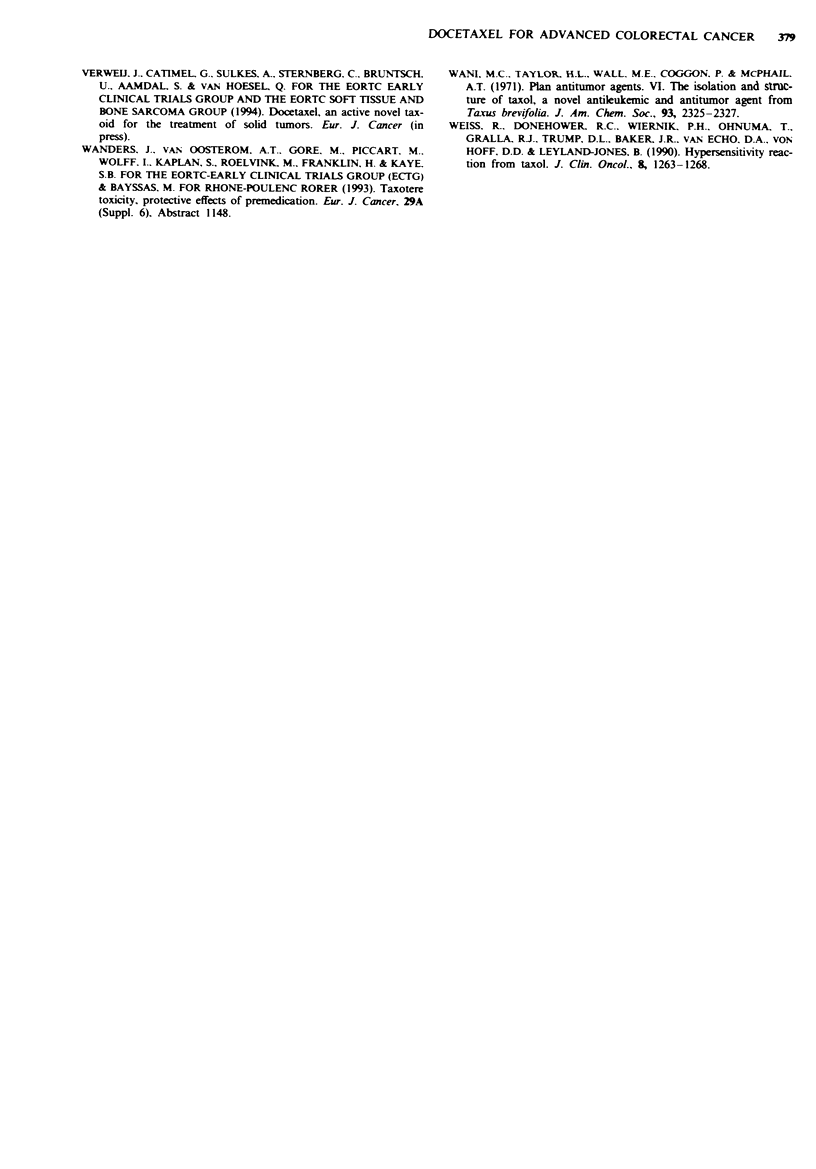# Docetaxel (Taxotere), a novel taxoid, in the treatment of advanced colorectal carcinoma: an EORTC Early Clinical Trials Group Study.

**DOI:** 10.1038/bjc.1994.309

**Published:** 1994-08

**Authors:** C. N. Sternberg, W. W. ten Bokkel Huinink, J. F. Smyth, V. Bruntsch, L. Y. Dirix, N. A. Pavlidis, H. Franklin, S. Wanders, N. Le Bail, S. B. Kaye

**Affiliations:** Regina Elena Cancer Institute, Rome, Italy.

## Abstract

Docetaxel (Taxotere), a new semisynthetic taxoid, is a potentially important chemotherapeutic agent for the treatment of cancer. Forty patients with bidimensionally measurable advanced adenocarcinoma of the colon were treated with docetaxel 100 mg m-2 every 3 weeks as a 1 h infusion without routine premedication. Thirty-nine patients were eligible: 23 males and 16 females. Median age was 60 years (range 41-75) and WHO performance status 1 (0-2). Prior adjuvant chemotherapy was performed in four patients and prior radiotherapy in nine patients. Bidimensionally measurable disease sites included: liver in 26 patients, lymph nodes and abdominal/peritoneal masses in 13, lung/mediastinal masses in ten and subcutaneous nodes in four. The median number of cycles given was 2 (range 1-15). Thirty-three patients were evaluable for response. One patient (3%) achieved a complete response and two (6%) (95% confidence limits 0-14%) a partial response. Side-effects were similar to those observed in other studies. Docetaxel, given at this dosage and schedule, has minimal activity in the treatment of colorectal carcinoma.


Br. J. Cancer (1994), 70, 376-379 © Macmillan Press Ltd., 1994

### Docetaxel (Taxotere™), a novel taxoid, in the treatment of advanced colorectal carcinoma: an EORTC Early Clinical Trials Group Study

C.N. Sternberg[1], W.W. Ten Bokkel Huinink[2], J.F. Smyth[3], V. Bruntsch[4], L.Y. Dirix[5], N.A. Pavlidis[6], H. Franklin[7], S. Wanders[7], N. Le Bail[8] & S.B. Kaye[9]

[1]*Regina Elena Cancer Institute, 00145 Rome, Italy;* [2]*Netherland Cancer Institute Antoni van Leeuwenhoekhuis, 1066 CX Amsterdam, The Netherlands;* [3]*Western General Hospital, EH4 2XU Edinburgh, UK;* [4]*5' Medizinische Klinik, 90340 Nurnberg, Germany;* [5]*Universitair Ziekenhuis, B-2520 Antwerp, Belgium;* [6]*University Hospital, 45110 Ioannina, Greece;* [7]*EORTC-NDDO, 1066 CX Amsterdam, The Netherlands;* [8]*Rhone Poulenc Rorer, 94403 Bitry Sur Seine Cedex, France;* [9]*Beatson Oncology Centre, 92165 Glasgow, UK.*

**Summary** Docetaxel (Taxotere), a new semisynthetic taxoid, is a potentially important chemotherapeutic agent for the treatment of cancer. Forty patients with bidimensionally measurable advanced adenocarcinoma of the colon were treated with docetaxel 100 mg m$^{-2}$ every 3 weeks as a 1 h infusion without routine premedication. Thirty-nine patients were eligible: 23 males and 16 females. Median age was 60 years (range 41–75) and WHO performance status 1 (0–2). Prior adjuvant chemotherapy was performed in four patients and prior radiotherapy in nine patients. Bidimensionally measurable disease sites included: liver in 26 patients, lymph nodes and abdominal/peritoneal masses in 13, lung/mediastinal masses in ten and subcutaneous nodes in four. The median number of cycles given was 2 (range 1–15). Thirty-three patients were evaluable for response. One patient (3%) achieved a complete response and two (6%) (95% confidence limits 0–14%) a partial response. Side-effects were similar to those observed in other studies. Docetaxel, given at this dosage and schedule, has minimal activity in the treatment of colorectal carcinoma.

Colorectal carcinoma is one of the most frequent malignancies in Europe and the second most common malignancy in the United States, with more than 150,000 new cases diagnosed in the United States and 60,000 deaths per year (Boring *et al.*, 1991). Surgical resection and more recently adjuvant chemotherapy have become the standard approach for early stage disease (Grem, 1991). However, approximately 20% of patients have metastases at the time of diagnosis, and nearly 50% of patients will eventually develop metastatic disease (Kemeny, 1987).

Systemic chemotherapy with 5-fluorouracil (5-FU)-based therapy has been standard treatment for the last 30 years with a response rate of 20% (Heiderberg *et al.*, 1957; Hansen, 1990). Newer therapeutic regimens are almost always compared with 5-FU. However, no meaningful survival differences have been demonstrated when compared with no treatment. Many 5-FU-containing combinations have been investigated with poor overall response rates ranging from 8% to 37% (Valone *et al.*, 1989). Recently, biochemical modulation of 5-FU with leucovorin has been shown to improve the overall response rates compared with 5-FU alone (Erlichman *et al.*, 1988; Petrelli *et al.*, 1989; Poon *et al.*, 1989; Doroshow *et al.*, 1990). Only few studies, however, have shown improvement in survival (Erlichman *et al.*, 1988; Petrelli, 1989; Hansen, 1990). A variety of other single agents have only occasionally produced some tumour regression (Coehn *et al.*, 1989; Bruckner, 1991). Since few truly effective treatment options are available for advanced colorectal carcinoma, new and more effective agents are urgently needed.

Paclitaxel (Taxol), extracted from the Pacific Yew *Taxus brevifolia* (Wani *et al.*, 1971), has been found to have significant activity in several human malignancies, including refractory ovarian cancer and breast cancer (McGuire *et al.*, 1989; Holmes *et al.*, 1991). Docetaxel (Taxotere) is a taxoid semisynthesised from a precursor extracted from the needles of the European yew, *Taxus baccata* (Mangatal *et al.*, 1989). This source, in contrast to the source of paclitaxel, is renewable, and docetaxel is formulated in polysorbate 80 instead of Cremophor EL, which is thought to be responsible for some of the side-effects of paclitaxel (Weiss *et al.*, 1990).

Docetaxel and paclitaxel both induce the formation of stable microtubule polymers and thus disturb the architecture of the cytoskeleton as well as the orderly progression through mitosis (Ringel, 1991; Horwitz, 1992). Docetaxel is twice as potent in the tubulin depolymerisation assay.

This mechanism of action differs from that of other spindle poisons such as vinca alkaloids, which inhibit tubulin assembly in microtubules. In preclinical testing, docetaxel was active against three murine colon tumours, C38, C51 and C26 (Geran *et al.*, 1972), and the human tumour xenograft CX-1 (Harrison *et al.*, 1992).

In phase I studies, mucositis and neutropenia were dose limiting. Mucositis was more frequently associated with longer infusion schedules. The highest dose intensity could be achieved with a 1 h infusion. The phase I study using this dose intensity showed a maximum tolerated dose of 115 mg m$^{-2}$, and the recommended dose was 100 mg m$^{-2}$ once every 3 weeks. We performed a phase II study using this dose and schedule for patients with advanced colorectal carcinoma.

### Patients and methods

Eligibility criteria included histologically or cytologically verified colorectal cancer with progression of disease. Eligible patients had to have locally advanced, unresectable or metastatic colorectal cancer. The presence of at least one bidimensionally measurable lesion was required. WHO performance status $<2$, life expectancy of $>12$ weeks, absolute neutrophils $>2{,}000$ ml$^{-1}$, platelets $>100{,}000$ ml$^{-1}$, age $>18$ years and $<75$ years, creatinine $<140$ mmol l$^{-1}$ (1.6 mg dl$^{-1}$) or a creatinine clearance $>60$ ml min$^{-1}$ bilirubin $<1.25\times$ upper normal limit, ASAT (SGOT) $<2\times$ the upper normal limit of $3\times$ in case of proven liver metastases were all necessary.

No prior chemotherapy was allowed with the exception of prior adjuvant chemotherapy, with a treatment-free interval of at least 1 year. A minimum of 4–8 weeks after prior radiotherapy was required; prior radiotherapy was not allowed if it included the sole marker lesion. In all patients written or oral informed consent was obtained.

Correspondence: C.N. Sternberg, Consultant Medical Oncologist, CTO Hospital, Via San Nemesio 21, 00145 Rome, Italy.
Received 28 February 1994; and in revised form 15 April 1994.

*Pharmaceutical data*

Docetaxel was provided as a sterile solution at a concentration of 40 mg $ml^{-1}$ in polysorbate 80 in 2 ml vials. Vials were stored at 4°C and protected from light. Immediately prior to use the solution was diluted in the vial by using 6 ml of either 5% dextrose or 0.9% saline. The solution was then immediately shaken for 20 s using a vortex mixer to obtain a clear solution. The appropriate amount of drug was then further diluted in either 5% dextrose or 0.9% saline to a maximum concentration of 1 mg $ml^{-1}$ docetaxel. This solution was administered i.v. over 1 h.

*Dosage and administration*

Each cycle of treatment consisted of docetaxel 100 mg $m^{-2}$ administered as an intravenous infusion over 1 h every 3 weeks. Each patient was scheduled to receive all cycles of treatment at the same dose. Doses were reduced for haematological and other toxicities. Adjustments were made according to the organ system showing the most severe toxicity. Toxicities were graded according to the common toxicity criteria (CTC). In the case of grade 4 neutropenia or thrombocytopenia, and also in case of grade 2 skin toxicity, the dose was reduced to 75% of the previous dose, i.e. to 75 mg $m^{-2}$ and 55 mg $m^{-2}$. If on treatment day 1 the neutrophil count was $<$1,500 μ$l^{-1}$ and platelets were $<$100,000 μ$l^{-1}$, the dose was delayed for 1 week and subsequent dose adjustment was made according to the nadir. Patients were removed from the protocol if there was no recovery after 1 week.

Routine use of antiemetics was not allowed. In addition, routine premedication was not used to prevent anaphylactoid or hypersensitivity reactions. For mild symptoms such as localised cutaneous reactions, pruritus and flushing the rate of infusion was decreased until symptoms regressed. For moderate symptoms such as generalised pruritus, flushing, rash, dyspnoea or hypotension, the docetaxel infusion was stopped and intravenous corticosteroids were given. For severe symptoms, such as bronchospasm, generalised urticaria, hypotension with systolic BP $<$80 mmHg or angio-oedema, the infusion was stopped and steroids and antihistamines were administered. Whenever possible docetaxel was resumed within 3 h after recovery or the patient was reinfused within 72 h using premedication. In case of severe hypersensitivity reactions all subsequent courses were preceded by a combination of dexamethasone and an antihistamine. Responses were classified according to WHO criteria and assessed every two courses. Treatment was continued until progression of disease or the occurrence of unacceptable side-effects.

### Results

*Patient characteristics*

Forty patients were entered into the study. Patient characteristics are described in Table I. Four patients had undergone prior adjuvant chemotherapy. All patients had bidimensionally measurable lesions.

*Response*

Thirty-three patients were evaluable for response. One patient was ineligible owing to concurrent use of corticosteroids. Six patients were inevaluable for response: three of these patients received only one dose of docetaxel and were not further evaluated for response. Of these three, two patients had excessive toxicity, one patient had grade 4 neutropenia and one patient had grade 2 headaches, fever, skin reaction and myelosuppression. The third patient had gastrointestinal bleeding. Another patient was not evaluable owing to violation of the protocol dose, and one patient did not complete the second cycle and died from sepsis. An additional patient died suddenly after two cycles prior to evaluation of his disease. No autopsy was performed.

**Table I** Patient characteristics of 39 eligible patients treated with docetaxel for colorectal cancer

| | |
|---|---|
| Entered | 40 |
| Ineligible | 1 |
| Evaluable for response | 33 |
| Male:female | 23:16 |
| Median age (range) (years) | 60 (41–75) |
| WHO performance status median (range) | 1 (0–2) |
| Prior surgery | 35 |
| Prior adjuvant chemotherapy | 4 |
| Prior immunotherapy | 1 |
| Prior radiotherapy | 9 |
| Bidimensionally measurable lesions | |
| Liver | 26 |
| Lymph nodes and abdominal peritoneal masses | 13 |
| Lung/mediastinal masses | 10 |
| Subcutaneous nodes | 4 |
| Median number of cycles (range) | 2 (1–15) |

**Table II** Side-effects (highest CTC grade per patient)

| | CTC grade | | | | |
|---|---|---|---|---|---|
| | *1* | *2* | *3* | *4* | *Total (%)* |
| *Non-haematological toxicity* | | | | | |
| Alopecia | 8 | 26 | | | 34 (87) |
| Fatigue | 8 | 14 | 5 | | 27 (69) |
| Skin | 6 | 18 | | | 24 (61) |
| Gastrointestinal | | | | | |
| Nausea | 12 | 3 | 2 | | 17 (43) |
| Vomiting | 6 | 5 | | | 11 (28) |
| Diarrhoea | 6 | 9 | 1 | | 16 (41) |
| Stomatitis | 9 | 6 | | | 15 (38) |
| Neurotoxicity | 9 | 4 | | 1 | 14 (36) |
| Hypersensitivity reactions | 7 | 1 | 3 | 2 | 13 (33) |
| Fever | 5 | 8 | | | 13 (33) |
| Headache | 5 | 4 | | | 9 (23) |
| Infections | 3 | 3 | 2 | | 8 (20) |
| Oedema | 2 | 3 | 1 | | 6 (15) |
| *Haematological toxicity* | | | | | |
| White blood cells | 3 | 19 | 13 | 3 | 38 (97) |
| Neutrophils | | 5 | 6 | 27 | 38 (97) |
| Platelets | 3 | | | | 3 (8) |

In one patient (3%) with liver metastases a complete response lasting 54 weeks was obtained, two patients (6%) (95% confidence limits 0–14%) attained a partial response (PR) in liver metastases, nine (27%) patients had stable disease and 21 (64%) progressed. The median time to progression in the evaluable patients was 1.5 months and the median survival was 7.5 months. A total of 118 cycles of docetaxel were administered. The median number of cycles per patient was 2 (range 1–15). Twenty-seven patients had no dose reduction. Twelve patients received 75 mg $m^{-2}$ in 26 cycles, and two of these patients subsequently also received 55 mg $m^{-2}$ docetaxel in three cycles. Reasons for dose reduction were haematological toxicity (seven cycles), non-haematological (mostly skin) toxicity (20 cycles) or both (two cycles). The most frequent reason for a patient to go off study was tumour progression.

*Toxicity*

The most important side-effects are described in Table II, based on 39 eligible patients. Alopecia was almost universal (87%). Grade 3–4 leucopenia occurred in 16 (41%) patients and grade 3–4 neutropenia in 85% of patients. Coinciding sepsis was reported in only one cycle; five patients required antibiotics for mild infections during neutropenia, each during one cycle. Mild to moderate skin toxicity was seen in 24 (61%) patients. This most commonly consisted of dry skin, erythema, pruritus, maculas, papulas and nail changes. Six

(15%) patients developed fluid retention, which was most often peripheral oedema. Mild to moderate neurosensory toxicity was observed in 13 patients (33%), neuromotor toxicity in only one. Fatigue occurred in 27 (69%) patients.

Hypersensitivity reactions were seen in 19 cycles (16%). These were mostly mild. The majority of hypersensitivity reactions occurred during cycle 1, within the first 5 min. Gastrointestinal side-effects included mild to moderate nausea in 17 (43%) patients, vomiting in 11 (28%), diarrhoea in 16 (41%), and mild to moderate stomatitis in 15 (38%).

### Discussion

The toxicities observed with docetaxel in this phase II study were similar to those observed in the treatment of patients with other solid tumours (Verweij *et al*., 1994). Reversible alopecia was practically universal and occurred within 2-4 weeks after the start of therapy. Hypersensitivity reactions, as have been observed with other taxoids, were relatively infrequent and were rarely severe despite the fact that no routine premedication was used. Premedication including corticosteroids and antihistamines further reduces the incidence of these reactions (Schrijvers *et al*., 1993). Other toxicities were similar to those observed with paclitaxel. Additional toxicities included a pruritic skin eruption and peripheral oedema. Skin toxicity consisted of erythema, desquamation and infrequent exfoliation or presented as nail toxicity with calcification and onycholysis. These toxicities were not reduced by the routine use of corticosteroids (Wanders *et al*., 1993). Fluid retention was most probably related to the cumulative dose of docetaxel, occurring infrequently at cumulative doses below 400 mg $m^{-2}$ (Wanders *et al*., 1993).

In phase II studies docetaxel has already been shown to have anti-tumour activity in breast cancer, non-small cell lung cancer, ovarian cancer, soft-tissue sarcoma, head and neck cancer, gastric cancer and melanoma (Verweij *et al*., 1994). No activity was seen in renal cancer (Bruntsch *et al*., 1993). Based upon the results of this study and the study of Pazdur *et al*. (1994), docetaxel, like paclitaxel, has minimal activity in the treatment of colorectal carcinoma. One mechanism conferring resistance to docetaxel and paclitaxel is the multidrug resistance P-glycoprotein, although the clinical relevance of this in colorectal cancer is uncertain (Lehnert *et al*., 1992). Treatment of advanced colorectal carcinoma will require further study with new agents in view of its insensitivity to most available cytotoxic drugs.

### References

BORING, C.C., SQUIRES, T.S. & TONG, T. (1991). *Cancer Stat*., **41**, 19-36.

BRUCKNER, H.W. & MOTWANI, B.T. (1991). Chemotherapy of advanced cancer of the colon and rectum. *Semin. Oncol*., **18**(5), 443-461.

BRUNTSCH, U., VAN OOSTEROM, A., HEINRICH, B., PARIDAENS, R., DE MULDER, P.H.M., WANDERS, J., FRANKLIN, H., BAYSSAS, M. & KAYE, S.B. (1993). Taxotere in advanced renal cell carcinoma. A phase II trial of the EORTC Early Clinical Trial Group. *Eur. J. Cancer*, **29A** (Suppl. 6), Abstract 1310.

COEHN, A.M., SHANK, B., FRIEDMAN, M.A. (1989). Colorectal cancer. In *Cancer Principles and Practice of Oncology*, 3rd edn, DeVita, Jr, V.J., Hellman, S. & Rosenberg, S. (eds) pp. 895-964. J.B. Lippincott: Philadelphia.

DOROSHOW, J.H., MULTHAUF, P., LEONG, L., MARGOLIN, K., LITCHFIELD, T., AKMAN, S., CARR, B., BERTRAND, M., GOLDBERG, D., BLAYNEY, D., ODUJINRIN, O., DELAP, R., SHUSTER, J. & NEWMAN, E. (1990). Prospective randomized comparison of fluorouracil versus fluorouracil and high dose continuous infusion leucovorin calcium for the treatment of advanced measurable colorectal cancer in patients previously unexposed to chemotherapy. *J. Clin. Oncol*., **8**, 491-501.

ERLICHMAN, C., PINE, S., WONG, A. & ELHAKIM, T. (1988). A randomized trial of fluorouracil and folinic acid in patients with metastatic colorectal carcinoma. *J. Clin. Oncol*., **6**, 469-475.

GERAN, R.I., GREENBERG, N.H., MACDONALD, M.M., SCHUMACHER, A.M. & ABBOT, B.J. (1972). Protocols for screening chemical agents and natural products against animal tumours and other biological systems. *Cancer Chemother. Rep*. Part 3, 1.

GREM, J.L. (1991). Adjuvant treatment of node positive colon carcinoma with levamisole and 5-fluorouracil. *Oncology*, **5**, 63-70.

HANSEN, R.M. (1990). Systemic therapy in metastatic colorectal cancer. *Arch. Intern. Med*., **150**, 2265-2269.

HARRISON, S.D., DYKES, D.J., BISSERY, M.C. & GRISWOLD, D.P. (1992). Response of four human tumor xenografts in nude mice to taxotere. *Ann. Oncol*., **3**, (Suppl. 1), 121 (Abstract 250).

HEIDERBERGER, C., CHAUDARI, N.K. & DANNENBERG, P. (1957). Fluorinated pyrimidine. A new class of tumour inhibitory compounds. *Nature*, **179**, 663-666.

HOLMES, F.A., FRYE, D., THERLAULT, H.L., WALTERS, R.S., FORMAN, A.D., NEWTON, L.K., BUZDAR, A.U. & HORTOBAGYL, G.N. (1991). Phase II study of taxol in patients with metastatic breast cancer. *Proc. Am. Soc. Clin. Oncol*., **10**, Abstract 113.

HORWITZ, S.B. (1992). Mechanism of action of taxol. *Trends Pharmacol. Sci*., **13**, 134-136.

KEMENY, N. (1987). Role of chemotherapy in the treatment of colorectal carcinoma. *Semin. Surg. Oncol*., **3**, 190-214.

LEHNERT, M., EMERSON, S., DALTON, W.S., SALMON, S.E., ARIZONA CANCER CENTER, TUCSON, AZ, USA, & MED. KLINIK C, ST. GALLEN, SWITZERLAND (1992). Reversal of p-glycoprotein-associated resistance to taxol and taxotere. *Ann. Oncol*., **3** (Suppl. 1), Abstract 059.

MCGUIRE, W.P., ROWINSKI, E.K., ROSENSHEIN, N.B., GRUMBINE, F.C., ETTINGER, D.S., ARMSTRONG, D.K., DONEHOWER, R.C. (1989). Taxol, a unique antineoplastic agent with significant activity in advanced ovarian epithelial neoplasms. *Ann. Intern. Med*., **111**, 273-279.

MANGATAL, L., ADELINE, M.T., GUENARD, D., GUERETTE VOEGELEIN, F. & POTIER, P. (1989). Application of the vicinal oxyamination reaction with asymmetric induction to the hemisynthesis of taxol and analogues. *Tetrahedron*, **45**, 4177-4190.

PAZDUR, R., LASSERE, Y., BREADY, B., SOH, L., SOO, E., AJAMI, J.A., SUGARMAN, S., PATT, Y., ABBRUZZEE, J.L. & RABER, M.N. (1994). Phase II trial of docetaxel in metastatic colorectal carcinoma. *Ann. Oncol*., **5** (Suppl. 5), 203 (Abstract 513).

PETRELLI, N., DOUGLASS, Jr, H.O., HERRERA, L., RUSSELL, D., STABLEIN, D.M., BRUCKNER, H.W., MAYER, R.J., SCHINELLA, R., GREEN, M.D., MUGGIA, F.M., MEGIBOW, A., GREENWALD, E.S., BUKOWSKI, R.M., HARRIS, J., LEVIN, B., GAYNOR, E., LOUTFI, A., KALSER, M.H., BARKIN, J.S., BENEDETTO, P., WOOLLEY, P.V., NAUTA, R., WEAVER, D.W. & LEICHMAN, L.P. FOR THE GASTROINTESTINAL TUMOR STUDY GROUP (1989). The modulation of fluorouracil with leucovorin in metastatic colorectal carcinoma: a propsective randomized phase III trial. *J. Clin. Oncol*., **7**, 1419-1428.

POON, M.A., O'CONNELL, M.J., MOERTEL, C.G., WIEAND, H.S., CULLINAN, A., EVERSON, L.K., KROOK, J.E., MAILLIARD, J.A., LAURIE, J.A., TSCHETTER, L.K. & WIESENFELD, M. (1989). Biochemical modulation of fluorouracil: evidence of significant improvement of survival and quality of life in patients with advanced colorectal carcinoma. *J. Clin. Oncol*., **7**, 1407-1481.

RINGEL, I. & HORWITZ, S.B. (1991). Studies with RP 56976 (taxotere): a semisynthetic analogue of taxol. *J. Natl Cancer Inst*., **83**, 288-291.

SCHRIJVERS, D., WANDERS, J., DIRIX, L., PROVE, A., VONCK, I. VAN OOSTEROM, A.T. & KAYE, S. (1993). Coping with toxicities of docetaxel (Taxotere). Short report. *Ann. Oncol*., **4**, 610-611

VALONE, F.H., FRIEDMAN, M.A., WITTLINGER, P.S., DRAKES, T., EISENBERG, P.D., MALEC, M., HANNIGAN, J.F. & BROWN, Jr, B.W. (1989). Treatment of patients with advanced colorectal carcinomas with fluorouracil alone, high dose leucovorin plus fluorouracil or sequential methotrexate, fluorouracil, leucovorin: a randomized trial of the Northern California Oncology Group. *J. Clin. Oncol*., **7**, 1427-1436.

VERWEIJ, J., CATIMEL, G., SULKES, A., STERNBERG, C., BRUNTSCH, U., AAMDAL, S. & VAN HOESEL, Q. FOR THE EORTC EARLY CLINICAL TRIALS GROUP AND THE EORTC SOFT TISSUE AND BONE SARCOMA GROUP (1994). Docetaxel, an active novel taxoid for the treatment of solid tumors. *Eur. J. Cancer* (in press).

WANDERS, J., VAN OOSTEROM, A.T., GORE, M., PICCART, M., WOLFF, I., KAPLAN, S., ROELVINK, M., FRANKLIN, H. & KAYE, S.B. FOR THE EORTC-EARLY CLINICAL TRIALS GROUP (ECTG) & BAYSSAS, M. FOR RHONE-POULENC RORER (1993). Taxotere toxicity, protective effects of premedication. *Eur. J. Cancer*, **29A** (Suppl. 6), Abstract 1148.

WANI, M.C., TAYLOR, H.L., WALL, M.E., COGGON, P. & MCPHAIL, A.T. (1971). Plan antitumor agents. VI. The isolation and structure of taxol, a novel antileukemic and antitumor agent from *Taxus brevifolia*. *J. Am. Chem. Soc.*, **93**, 2325–2327.

WEISS, R., DONEHOWER, R.C., WIERNIK, P.H., OHNUMA, T., GRALLA, R.J., TRUMP, D.L., BAKER, J.R., VAN ECHO, D.A., VON HOFF, D.D. & LEYLAND-JONES, B. (1990). Hypersensitivity reaction from taxol. *J. Clin. Oncol.*, **8**, 1263–1268.